# Xeno-Free Integrated Platform for Robust Production of Cardiomyocyte Sheets from hiPSCs

**DOI:** 10.1155/2022/4542719

**Published:** 2022-11-23

**Authors:** Tiago P. Dias, Tânia Baltazar, Sandra N. Pinto, Tiago G. Fernandes, Fábio Fernandes, Maria Margarida Diogo, Manuel Prieto, Joaquim M. S. Cabral

**Affiliations:** ^1^iBB—Institute for Bioengineering and Biosciences and Department of Bioengineering, Instituto Superior Técnico, Universidade de Lisboa, Av. Rovisco Pais, 1049-001 Lisboa, Portugal; ^2^Associate Laboratory i4HB—Institute for Health and Bioeconomy at Instituto Superior Técnico, Universidade de Lisboa, Av. Rovisco Pais, 1049-001 Lisboa, Portugal

## Abstract

Human induced pluripotent stem cells (hiPSCs) can be efficiently differentiated into cardiomyocytes (CMs), which can be used for cardiac disease modeling, for drug screening, and to regenerate damaged myocardium. Implementation of xeno-free culture systems is essential to fully explore the potential of these cells. However, differentiation using xeno-free adhesion matrices often results in low CM yields and lack of functional CM sheets, capable of enduring additional maturation stages. Here, we established a xeno-free CM differentiation platform using TeSR/Synthemax, including a replating step and integrated with two versatile purification/enrichment metabolic approaches. Results showed that the replating step was essential to reestablish a fully integrated, closely-knit CM sheet. In addition, replating contributed to increase the cTnT expression from 65% to 75% and the output from 2.2 to 3.1 CM per hiPSC, comparable with the efficiency observed when using TeSR/Matrigel. In addition, supplementation with PluriSin1 and Glu^−^Lac^+^ medium allowed increasing the CM content over 80% without compromising CM sheet integrity or functionality. Thus, this xeno-free differentiation platform is a reliable and robust method to produce hiPSC-derived CMs, increasing the possibility of using these cells safely for a wide range of applications.

## 1. Introduction

Cardiovascular diseases (CVDs) are the number one cause of deaths worldwide, with 17.9 million related deaths in 2019 corresponding to 32% of all deaths globally, as estimated by the World Health Organization [1]. From these, 85% were caused by heart attack or stroke. In a severe ischemic event, the heart left ventricle can lose up to 10^9^ billion cardiomyocytes (CMs) [2]. Moreover, cardiac tissue presents a very low regenerative capacity [3, 4]. Thus, cardiac muscle regeneration after a severe heart attack requires an external source of CMs.

Human induced pluripotent stem cells (hiPSCs) can give rise to a wide range of functional cells with enormous potential for regenerative medicine applications [5]. CMs are among the most promising hiPSC-derived cells and can be used for disease modeling, for drug screening and as a cell source to treat damaged cardiac tissues [6–8]. In the last years, multiple clinical trials have been initiated either using human embryonic stem cell (hESC)-derived cardiac progenitors [9] or hiPSC-derived cardiomyocytes (hiPSC-CMs) [10] (NCT04396899, NCT03763136, and NCT03759405), mainly relying on engineered cardiac patches or cell sheets. However, for the safe and standard clinical implementation of these therapies, optimization of culture conditions, free of animal-derived components, and their integration with purification steps are of utmost importance.

Maintenance and differentiation of hiPSCs is commonly performed using Matrigel (or Geltrex), an undefined mixture of basement membrane ECM proteins extracted from Engelbreth-Holm-Swarm mouse tumors. The quality and composition of these undefined matrices can vary from lot to lot and present safety concerns for clinical applications due to the risk of contamination with animal-derived pathogens and immunogens [11, 12]. Chemically-defined and xeno-free matrices, such as vitronectin [13, 14], Synthemax [15]—a vitronectin peptide—and laminin [16], can be used as alternatives. However, hiPSC cardiac differentiation induces a shift in integrin expression, which commonly leads to cellular detachment from this type of chemically-defined matrices [17–19]. Thus, strategies to mitigate cellular detachment during hiPSC cardiac differentiation in xeno-free culture systems are needed.

In addition, cell therapies based on the use of human pluripotent stem cell (hPSC) derivatives have the associated risk of teratoma formation. Thus, it is vital to integrate efficient differentiation platforms with purification steps to selectively eliminate potential undifferentiated hiPSCs that may remain in culture. Moreover, purification and enrichment steps that promote cell sheet integrity while fine-tuning the CM percentage are essential to allow further integration with maturation processes, important for drug screening and disease modelling applications [20]. A variety of methods, such as FACS, MACS, or miRNA-responsive mRNA constructs, have been described to be effective in purifying and/or enriching hiPSC-derived cell cultures [21–26]. However, these methods require disruption of the cell sheet or genetic manipulation of hiPSCs. An alternative is to use metabolic purification/enrichment strategies, which selectively eliminate hiPSCs by exploring metabolic differences between hiPSCs and hiPSC-derived cells. This provides multiple advantages over other methods since this methodological approach allows seamless integration with differentiation protocols. Ben-David et al. showed that hiPSCs can efficiently be eliminated using PluriSin1, a Stearoyl-CoA desaturase-1 (SCD1) inhibitor, without significantly affecting mature cells [27]. Likewise, Tohyama et al. reported a method to purify cardiac cultures, enhancing CM percentage and eliminating other cells, including hiPSCs, by removing glucose from the culture medium and replacing it with lactate [28].

Here, we describe the integration of a xeno-free hiPSC cardiac differentiation platform, based in the previously established WNT signaling modulation protocol, with a tunable two-step metabolic purification/enrichment phase, using PluriSin1 and glucose-free medium supplemented with lactate. Hurdles associated with severe cell detachment when using xeno-free coatings were overcome by using a replating step, which increased CM yield and reestablished CM sheet structural integrity. The hiPSC-CMs derived under xeno-free conditions showed a degree of maturation in-line with previously published results [20] and the conjugation of the replating step with the two metabolic purification/enrichment steps did not negatively impact hiPSC-CM functionality.

## 2. Materials and Methods

### 2.1. Human Induced Pluripotent Stem Cell Culture

In this work, the hiPSC cell line iPS-DF6-9-9 T.B, purchased from WiCell Bank, was used. This cell line is vector free and was derived from foreskin fibroblasts with a karyotype 46, XY. Maintenance of hiPSC culture was performed using mTeSR1 medium (STEMCELL Technologies) in 6-well tissue culture plates coated with Matrigel (BD Biosciences) diluted 1 : 30 in DMEM/F12. Prior to differentiation, hiPSCs were adapted in the same xeno-free culture system used for differentiation by using TeSR2 medium (STEMCELL Technologies) or Essential 8 medium (Thermo Fisher Scientific). Plates were coated with Synthemax II-SC (Corning), a synthetic vitronectin-based peptide, at a concentration of 5 *μ*g/cm^2^, Vitronectin XF (STEMCELL Technologies), a defined human recombinant protein, at a concentration of 1.25 *μ*g/cm^2^ or laminin from murine origin (Sigma) at a concentration of 2.5 *μ*g/cm^2^. Enzyme-free passaging was performed using EDTA (Thermo Fisher Scientific) solution diluted in PBS at a concentration of 0.5 mM. Cells were incubated for 5 min with EDTA at room temperature and flushed with culture medium. For cell counting, a sample of 100 *μ*L was incubated in 400 *μ*L of Accutase for 7 min at room temperature and samples were diluted in trypan blue. Phase contrast images were obtained using a Leica DMI 3000B microscope and a digital camera Nikon DXM 1200.

### 2.2. Human iPSC-Cardiomyocyte Differentiation, Replating and Purification

Human iPSCs were seeded at a density of 1 × 10^5^ cells/cm^2^ and maintained in pluripotent state conditions with daily medium change. At around 95% confluency, hiPSC cardiac differentiation was induced by adapting the WNT signaling modulation protocol previously described [29]. WNT signaling modulation was achieved by using CHIR99021, which activates WNT signaling by inhibiting GSK3-mediated *β*-Catenin degradation, and using IWP4, which inhibits WNT secretion and activity by preventing WNT palmitoylation by porcupine. For the replating step, cells were detached from the plate at day 6 using EDTA and RPMI/B27 minus insulin medium. Cells were replated in plates coated with vitronectin or Synthemax at ratios from 1 : 1 to 1 : 3. The purification step was performed by supplementing RPMI/B27 minus insulin medium at day 6 with 20 *μ*M of PluriSin1 (Cayman). For purification/enrichment, RPMI/B27 medium was changed at day 12 to RPMI without glucose (Thermo Fisher Scientific) supplemented with 4 mM of Sodium L-Lactate (Sigma).

CM output was calculated according to the equation:

(1) $$\begin{array}{c} \text{Cardiomyocyte Output}=\frac{{\left[\text{Cells}\right]}_{f}\times cTnT\%}{{\left[\text{Cells}\right]}_{i}}, \end{array}$$

in which [Cells]_*f*_ corresponds to the total number of viable cells counted at day 15, using the trypan blue method, which was multiplied by the percentage of cells expressing cTnT to obtain the number of final CMs and divided by [Cells]_*i*_, which corresponds to the number of initially seeded hiPSCs. The final CM numbers obtained upon the process of replating correspond to the sum of the CMs obtained in all splits originated from the initial replated well.

### 2.3. Flow Cytometry

Cells were washed with PBS and cardiac sheets were singularized using 0.25% (*w*/*v*) trypsin-EDTA (Thermo Fisher Scientific). Trypsin-EDTA was neutralized using RPMI/20% FBS medium. Cells were fixed using 2% (*w*/*v*) paraformaldehyde (PFA) for 20 min at room temperature. Then, cells were centrifuged and resuspended in 90% (*v*/*v*) cold methanol, incubated for 15 min at 4°C. Samples were then washed 3 times using a solution of 0.5% (*v*/*v*) BSA in PBS (FB1). Monoclonal mouse IgG primary antibody against cardiac troponin T (cTnT) (Thermo Scientific, Clone 13-11) was diluted at 1 : 250 in FB1 plus 0.1% (*v*/*v*) Triton (FB2) and incubated for 1 h at room temperature. Cells were then washed with FB2 and cell pellet resuspended with goat anti-mouse alexa-488 secondary antibody (Thermo Fisher Scientific) diluted 1 : 1000 in FB2 and incubated for 30 min in the dark. Cells were washed twice, and cell pellets were resuspended in 500 *μ*L of PBS and analyzed in a FACSCalibur flow cytometer (Becton Dickinson). Data were analyzed using FlowJo (v10, FlowJo LLC).

### 2.4. Real-Time PCR

RNA from each condition and controls was extracted using the High Pure RNA Isolation Kit (Roche) following manufacturer's instructions. RNA was quantified using a nanodrop and 1 *μ*g of RNA was converted to cDNA using the High-Capacity cDNA Reverse Transcription Kit (Thermo Fisher Scientific). Relative gene expression was evaluated using 10 ng of cDNA, 250 nM of each primer (Table S1) and using the Fast SYBR Green Master Mix (Thermo Fisher Scientific) with an annealing temperature set to 60°C. Melting curves were performed at the end to assess if primers were amplifying only the correct amplicon. Values were treated following the 2^-*ΔΔ*CT^ method. *GAPDH* gene expression was used as endogenous control and relative expression was calibrated using day 0 of differentiation.

### 2.5. Immunofluorescence Staining and Confocal Microscopy

Samples for confocal microscopy were prepared by replating hiPSC-CMs in coverslips (no.1, VWR) inside 24-well plate wells. After 24 h, cells were fixed with 4% (*w*/*v*) PFA for 15 min, washed with PBS and incubated with blocking solution (10% (*v*/*v*) NGS, 0.1% (*v*/*v*) Triton-X in PBS) for 1 h. After incubation, cTnT mouse IgG antibody (Thermo Scientific, Clone 13-11) was diluted 1 : 250 in staining solution (5% (*v*/*v*) NGS, 0.1% (*v*/*v*) Triton-X in PBS) and incubated for 2 h at room temperature. After washing with PBS, secondary antibody goat anti-mouse IgG Alexa-488 (Thermo Fisher Scientific) was diluted 1 : 500 in staining solution and incubated for 1 h at room temperature. Cells were then washed 2 times with PBS, incubated for 2 min with 3 *μ*g/mL of DAPI diluted in PBS and washed again 3 times. Then, the CMs in coverslips were covered by a lamella (no.1, VWR), hydrated with antifade mounting medium (Vectashield, Vector Labs) and sealed with varnish.

Confocal Scanning Laser Microscopy (CSLM) and two photon excitation images were acquired using a Leica TCS SP5 confocal inverted microscope (DMI6000) with a 63.3 x water-immersion apochromatic objective (1.2 numerical aperture). Alexa-488 excitation was performed using a 488 nm line of an Argon ion laser, and fluorescence emission was collected between 500 nm and 560 nm using the tunable system and beam splitter of the Leica TCS SP5. DAPI excitation was achieved using multiphoton excitation with a Ti:sapphire laser (Spectra-Physics Mai Tai BB, 710 – 990 nm) set to 780 nm as the excitation source. DAPI fluorescence emission was collected between 400 nm and 478 nm. Laser light intensity was controlled by an acoustic-optical filter system. Images were processed using ImageJ/Fiji (http://fiji.sc) [30]. Thin filament (cTnT) length measurement was performed using custom-written software, as previously described [20].

### 2.6. Calcium Imaging

Cells were replated on Matrigel-coated 8-well *μ*-slides (Ibidi) using EDTA. After 48 h, calcium transients were measured using the Fluo-4 Direct Calcium Assay kit (Thermo Fisher Scientific). Fluo-4 was prewarmed at 37°C and loaded into the cells by adding an equal volume to the culture medium present in the well. Cells were incubated at 37°C for 30 min. In experiments, using chemical receptor agonists, isoproterenol hydrochloride (Sigma) or carbamoylcholine chloride (Sigma) were diluted in prewarmed Fluo-4 solution and loaded into cells at a final concentration of 1 *μ*M. Cells were incubated at 37°C for 15 to 30 min.

Samples were measured using the confocal microscope previously described with a dry objective 10.0 × magnification (numerical aperture of 0.40), with images obtained at a resolution of 128 × 128 pixels, with a scan speed of 700 Hz, and with frame intervals of 97 msec during 60-120 sec. Fluo-4 excitation was performed using a 488 nm line with an Argon ion laser and fluorescence emission was collected (500-650 nm) using a Leica HyD hybrid detector. Samples were measured within 4-5 min after leaving the incubator using a heating stage set at 37°C to maintain the temperature before and during measurements.

### 2.7. Data and Statistical Analysis

Data and statistical analysis were performed using ANOVA to assess general differences among multiple data groups and unpaired two-sided Welch's *t*-tests to specifically compare two groups. *p* values lower than 0.05 were regarded as statistically significant (^∗^*p* value<0.05, ^∗∗^*p* value<0.01, ^∗∗∗^*p* value<0.001).

## 3. Results

### 3.1. The Effect of WNT Signaling Activation Coupled with Defined Adhesion Substrates on Cardiomyocyte Yield and Sheet Integrity

**Figure 1:**
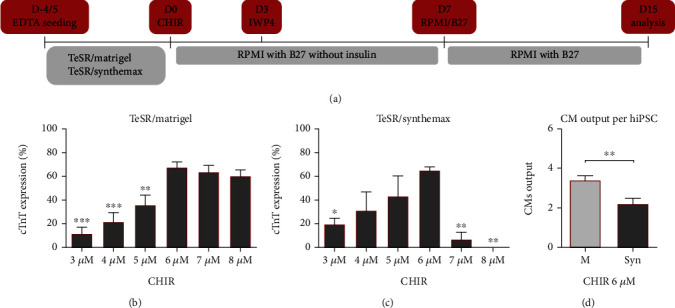
Optimization of hiPSC cardiac differentiation by WNT signaling modulation using different culture systems. (a) Cardiomyocyte differentiation protocol adapted from Lian et al. [29] using enzyme-free passaging and seeding. WNT signaling activation using CHIR was performed for 24 h, while inhibition using IWP4 was performed for 48 h. (b) Effect of CHIR concentration on cardiomyocyte differentiation efficiency in the culture system TeSR/Matrigel by measuring the cardiomyocyte marker cTnT by flow cytometry at day 15. Error bars, SEM: *n* = 5 for 3, 4, and 7 *μ*M, *n* = 6 for 5 and 8 *μ*M, *n* = 12 for 6 *μ*M. ^∗∗^*p* value<0.01, ^∗∗∗^*p* value<0.001 relative to 6 *μ*M (ANOVA with Tukey's test). (c) Screening of CHIR concentration impact on cardiomyocyte differentiation efficiency in the culture system TeSR/Synthemax measured by flow cytometry at day 15. Error bars, SEM: *n* = 3 for 3, 4, and 5 *μ*M, *n* = 2 for 7 and 8 *μ*M, *n* = 7 for 6 *μ*M. ^∗^*p* value<0.05, ^∗∗^*p* value<0.01 relative to 6 *μ*M (ANOVA with Tukey's test). (d) Cardiomyocyte (CM) output at day 15 per seeded hiPSC for each system using 6 *μ*M of CHIR. Error bars, SEM: *n* = 8 for TeSR/Matrigel (M), *n* = 7 for TeSR/Synthemax (Syn). ^∗∗^*p* value<0.01 (Welch's *t*-test).

We began by adapting the cardiac differentiation protocol from Lian et al. [29] to each culture system and specific model hiPSC line being used in this work, by optimizing the step of WNT signaling activation. In fact, this is a critical factor for cardiac differentiation reproducibility, since different hiPSC lines can present diverse levels of endogenous WNT signaling components and variations across cell-cycle profiles, which can result in inconsistent responses to culture conditions and signaling modulators [31]. For this purpose, we screened the impact on differentiation efficiency of the initial concentration of CHIR99021 (CHIR) by measuring the expression of the cardiomyocyte marker cTnT at day 15 of differentiation, using flow cytometry (Figure 1(a)). For all conditions, concentrations of CHIR over 8 *μ*M led to toxicity and cellular death/detachment. Moreover, the first 7 days of commitment were marked by an increased cell death, reshaping, and cell sheet reorganization (Video S1).

For both culture systems, TeSR/Matrigel and TeSR/Synthemax, 6 *μ*M of CHIR revealed to be the optimal concentration for CM differentiation, resulting in an average of 67% and 65% of cTnT positive cells, respectively (Figures 1(b) and 1(c)). For the system TeSR/Matrigel, 6 *μ*M of CHIR lead to the formation of closely-knit cardiac sheets (Video S2), while 7 *μ*M and 8 *μ*M often resulted in looser sheets. For TeSR/Synthemax, 7 *μ*M and 8 *μ*M of CHIR resulted in a reduced number or absence of cells with CM phenotype. In addition, one of the major challenges upon using the xeno-free system TeSR/Synthemax was the severe detachment of the cell sheet, after exposure to the chemical inhibitors, mainly when differentiation efficiencies were high (Video S3). This result was also observed when vitronectin or laminin were used (Figure S1A and S1B). Moreover, substitution of TeSR for E8 resulted in similar differentiation efficiency when Matrigel was used (Figure S1C). However, a very significant decrease of differentiation efficiency was observed when Matrigel was substituted by Synthemax, resulting in an average of 6.4% cTnT positive cells when 6 *μ*M of CHIR was used (Figure S1D). Consequently, due to CM and overall sheet detachment, differentiation yield for TeSR/Synthemax was significantly reduced, resulting in 2.2 CMs per seeded hiPSC compared with 3.4 for the TeSR/Matrigel culture system (Figure 1(d)).

### 3.2. Replating Reestablished Cardiac Sheets Integrity in Xeno-Free Conditions and Improved Cardiomyocyte Output

**Figure 2:**
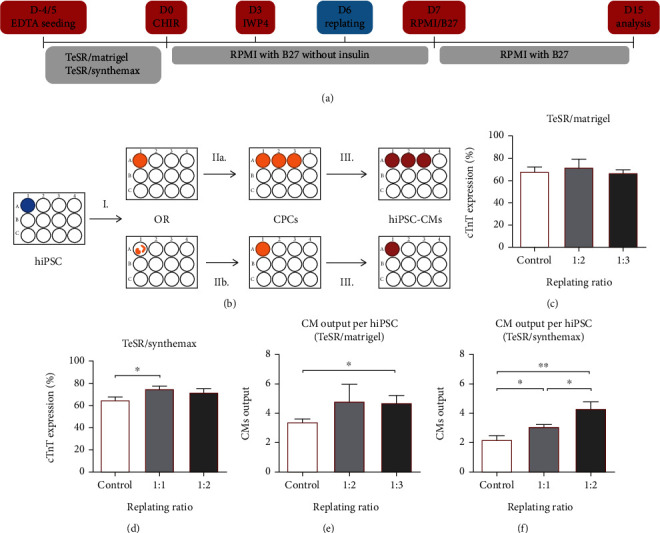
Replating step increased cell sheet integrity and cardiomyocyte output. (a) Modification of the WNT signaling modulation protocol by adding a replating step at day 6. (b) After initial WNT modulation steps (I.), addition of the replating step increased CM sheet output (IIa.) and for xeno-free systems (e.g., TeSR/Synthemax) reestablished the CM sheet after severe detachment (IIb.) by promoting the proliferation of cardiac progenitor cells (CPCs) (III.). (c) Replating ratios of 1 to 2 and 1 to 3 had no significant impact on differentiation efficiency in the culture system TeSR/Matrigel compared with differentiation without replating (Control). Error bars, SEM: *n* = 12 for Control (6 *μ*M) and 1 : 3, *n* = 5 for 1 : 2. (d) On the other hand, reestablishing the cell sheet (ratio 1 : 1) in TeSR/Synthemax provided an increase in differentiation efficiency compared with differentiation without replating (Control). Error bars, SEM: *n* = 7 for Control (6 *μ*M) and 1 : 2, *n* = 10 for 1 : 1. ^∗^*p* value<0.05 (Welch's *t*-test). (e) CM output at day 15 per seeded hiPSC increased with replating for the system TeSR/Matrigel. Error bars, SEM: *n* = 8 for Control (6 *μ*M) and 1 : 2, *n* = 11 for 1 : 3. ^∗^p value<0.05 (Welch's *t*-test). (f) CM output at day 15 per seeded hiPSC increased significantly with the replating step by reestablishing the cell sheet for the system TeSR/Synthemax. Error bars, SEM: *n* = 7 for Control (6 *μ*M), *n* = 6 for 1 : 1, *n* = 10 for 1 : 2. ^∗^*p* value<0.05, ^∗∗^*p* value<0.01 (Welch's *t*-test).

To address the problem of cell detachment when xeno-free coatings were used, we hypothesized that a replating step could be combined with the differentiation protocol to redistribute the cells prior to the occurrence of detachment events. Since inhibition of WNT signaling increased gene expression of *ISL1* and *NKX2.5*, we anticipated the appearance of cardiac progenitor cells (CPCs) in culture by day 5 (Figure S1E and S1F). Therefore, a replating step was integrated in the culture process at day 6 of differentiation (Figure 2(a)) since we hypothesized that CPCs would be the best population to maximize the benefit of the replating step due to the CPCs recognized proliferative capacity and cardiac multipotency [32]. This step would potentially allow the increase of the number of cardiac sheets, CM output and the reestablishment of the CM sheet when xeno-free matrices were used (Figure 2(b)).

For the system TeSR/Matrigel, the incorporation of a replating step at a split ratio of 1 : 2 or even 1 : 3 did not significantly affect differentiation efficiency (Figure 2(c)). On the other hand, for TeSR/Synthemax, a split ratio of 1 : 1 reestablished the cell sheet and resulted in a statistically significant increase from 65% to 75% of cTnT expression at day 15 compared with the control without replating (Figure 2(d)). Similar impact was observed for E8/Matrigel (Figure S1G). In addition, replating allowed a significant increase of CM output per seeded hiPSC for both systems, with an increase, for TeSR/Matrigel, from 3.4 to 4.8 or 4.7 (Figure 2(e)), if replating was performed at 1 : 2 or 1 : 3, respectively, and with an increase, for TeSR/Synthemax, from 2.2 to 3.1 or 4.3 (Figure 2(f)), if cells were replated at 1 : 1 or 1 : 2, respectively. A similar increase in output was observed for E8/Matrigel, from 3.4 to 4.8 (Figure S1G), if replating was performed at 1 : 2. Thus, overall, the introduction of a replating step allowed the reestablishment of the cardiac sheet under xeno-free culture conditions, and significantly increased the CM output.

### 3.3. Integration of a Two-Phase Purification and Enrichment Protocol

In addition to replating, two steps of purification and enrichment were integrated in the differentiation platform with the objective of eliminating the potentially remaining undifferentiated hiPSCs and also as a tunable tool to control the percentage of CMs in the sheets without damaging sheet structural integrity, which is essential for long-term maturation [20]. This was accomplished by supplementation of the culture medium with Plurisin1, which specifically eliminates hiPSCs by inhibiting oleic acid biosynthesis [27, 33], conjugated with the use of a glucose-free medium supplemented with 4 mM of Lactate (Glu^−^Lac^+^), which takes advantage of the biochemical differences of CMs compared with other cell types, in particular hiPSCs, to efficiently metabolize lactate to produce energy via oxidative phosphorylation [28].

**Figure 3:**
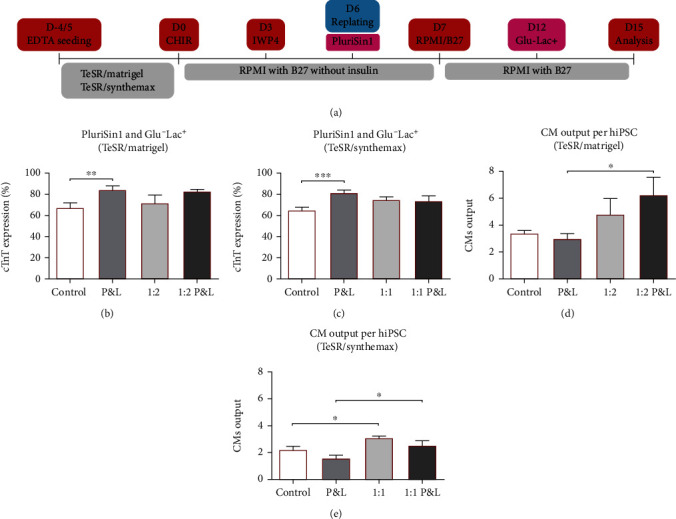
Integration in the differentiation platform of a two-phase purification and enrichment step. (a) Addition of a purification step using 20 *μ*M of PluriSin1 at day 6 and a purification/enrichment step at day 12 using glucose-free medium supplemented with lactate (Glu^−^Lac^+^). (b) Simultaneous introduction of the two-step Plurisin1 and Glu^−^Lac^+^ (P&L) increased differentiation efficiency in the system TeSR/Matrigel when compared with control. Error bars, SEM: *n* = 12 for Control (6 *μ*M), *n* = 7 for P&L, *n* = 5 for 1 : 2, *n* = 6 for 1 : 2 P&L. ^∗∗^*p* value<0.01 (Welch's *t*-test). (c) Similar impact of Plurisin1 and Glu^−^Lac^+^ was observed for the system TeSR/Synthemax when compared with control. Error bars, SEM: *n* = 7 for Control (6 *μ*M) and P&L, *n* = 10 for 1 : 1, *n* = 6 for 1 : 1 P&L. ^∗∗∗^*p* value<0.001 (Welch's *t*-test). (d) CM output at day 15 per seeded hiPSC for the system TeSR/Matrigel was not impacted significantly by P&L unless when replating was performed. Error bars, SEM: *n* = 8 for Control (6 *μ*M) and 1 : 2, *n* = 6 for P&L and 1 : 2 P&L. ^∗^*p* value<0.05 (Welch's *t*-test). (e) Similarly, CM output for the system TeSR/Synthemax was not impacted significantly by P&L unless replating was performed. Error bars, SEM: *n* = 7 for Control (6 *μ*M) and P&L, *n* = 6 for 1 : 1 and 1 : 1 P&L. ^∗^*p* value<0.05 (Welch's *t*-test).

Both PluriSin1 and Glu^−^Lac^+^ strategies, when used independently, successfully eliminated established hiPSC colonies in less than 48 h (Figure S2A). Use of a single or a double dose of 20 *μ*M of PluriSin1 did not show an impact on cTnT percentage on cultures without replating (Figure S2B), although double dosage often led to cell death when replating was performed (Figure S2C). On the other hand, Glu^−^Lac^+^ had a time-dependent impact on differentiation without replating (Figure S2D), although longer exposures than 48 h led to increased cell death and loss of CM sheet structural integrity, independently of the coating material being used (Figure S2F). With replating, although a decrease in cTnT expression was not evident (Figure S2E), cell death and cell sheet detachment were already observed at 48 h with a significant impact on CM output (Figure S2G). Therefore, the step of purification using Plurisin1 was integrated in the culture platform at day 6, concomitantly with replating, to maximize hypothetical hiPSCs exposure to the drug. Moreover, purification/enrichment with Glu^−^Lac^+^ was performed during 48 h for cultures without replating and during 24 h for replated cultures, at day 12, after a beating phenotype was observed, which confirmed the presence of CMs able to withstand the impact of glucose-free media and the use of lactate as a source of energy (Figure 3(a)).

Conjugation of both purification steps, using PluriSin1 and Glu^−^Lac^+^ (P&L), resulted in a significant increase in cTnT positive cells for both culture systems TeSR/Matrigel and TeSR/Synthemax, while the increase was not statistically significant for replated cultures, possibly due to a potential phenomenon of population selection provided by the replating step (Figures 3(b)–3(c)). For TeSR/Matrigel, P&L resulted in an increase of the percentage of cTnT positive cells from 67%, in the control, to 85% (Figure 3(b)). For TeSR/Synthemax, P&L resulted in an increase from 65%, in the control, to 82% (Figure 3(c)). In addition, the process of replating conjugated with P&L resulted in a significant increase in CM output for both systems (Figures 3(d) and 3(e)). For TeSR/Matrigel, CM output increased from 3.0 in P&L to 6.3 in P&L with replating at a split ratio of 1 : 2 (Figure 3(d)). For TeSR/Synthemax, CM output increased from 1.6 in P&L to 2.6 in P&L with replating at 1 : 1 (Figure 3(e)). Similar results were observed for E8/Matrigel (Figure S2H‑I). The impact of performing culture without replating (Video S4) and with replating (Video S5), with or without the P&L step, on cardiac sheet integrity can easily be observed by the naked eye.

### 3.4. Assessment of hiPSC-Derived Cardiomyocyte Structural Maturity and Functionality

**Figure 4:**
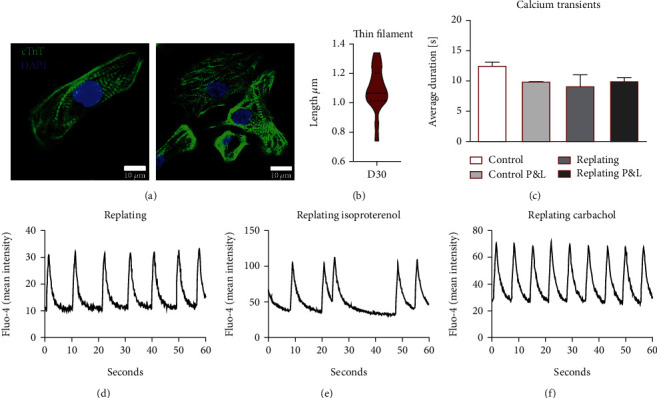
Structural and calcium transient analysis of hiPSC-derived cardiomyocytes. (a) Analysis of hiPSC-CM cardiac fibers organization at day 30 by confocal microscopy. A mixed population was observed. (b) Thin Filament length (cTnT bands) measured at day 30. Violin plot displays median and quartiles, *n* = 17 cardiomyocytes from two independent experiments. (c) Calcium transients measured at day 20 showed a similar average duration for all differentiation conditions. Error bars, SEM: *n* = 8 Control, *n* = 3 Control, P&L, and replating, *n* = 4 replating and P&L (ANOVA). (d) Representative calcium transient profile for replating, showing an average time between transients of 9.3, SD ± 0.9. (e) When 1 *μ*M isoproterenol was supplemented, arrhythmic behavior was observed for a few samples, with some cardiac sheets showing arrested contractibility and calcium transients. Figure shows an average time between calcium transients of 11.6, SD ± 8.6. (f) When 1 *μ*M carbachol was used, calcium transients showed a more consistent and synchronized pattern. Figure shows an average time between calcium transients of 6.9, SD ± 0.2.

Finally, CMs were structurally and functionally analyzed at day 30 of culture. A mixed population of CMs with aligned and nonaligned cardiac fibers was observed (Figure 4(a)). Thin filament length measured at day 30 was on average 1.09 *μ*m (Figure 4(b)), which is in line with results previously published by our group on the long-term maturation of hiPSC-CM sheets [20]. Introduction of the replating and P&L steps showed no statistically significant impact on calcium transient profiles (Figure 4(c)). A representative calcium profile obtained upon CMs replating can be observed in Figure 4(d). Response to isoproterenol, a *β*-adrenergic receptor agonist that increases heartbeat frequency, resulted in contractibility shutdown for most of the samples. Although some cell sheets maintained a beating phenotype, they showed an arrhythmic behavior, as exemplified in Figure 4(e), which highlights, as expected, the immaturity of the hiPSC-CMs at this stage of the maturation process [20]. Addition of carbachol, a muscarinic agonist that slows contraction frequency, did not completely revert the shutdown of the beating phenotype. Moreover, when carbachol was used first, without isoproterenol, a significant decrease in frequency was not observed. Overall, these results show no significant differences in terms of CM maturation between CMs obtained with and without replating and with or without purification/enrichment steps. As expected, at this stage of the differentiation process, some degree of structural and functional immaturity is still present.

In summary, these results demonstrate the successful development of a culture platform for the differentiation of hiPSC-CMs under xeno-free conditions that combines a replating step, to reestablish the cardiac sheet, with tunable tools for hiPSC depletion and cardiomyocyte enrichment while maintaining cell sheet integrity, essential for further hiPSC-CMs long-term maturation, and CM functionality.

## 4. Discussion

In recent years, potential therapies relying on hiPSC-CMs and cardiac progenitors have been steered towards clinical studies. However, the lack of efficient xeno-free differentiation platforms integrated with methodologies to selectively eliminate undifferentiated hiPSCs can hinder the safety of these therapeutic approaches. The focus of this work was to establish and optimize a xeno-free cardiac differentiation platform integrated with purification/enrichment steps. First, we optimized the cardiac differentiation process using Synthemax, a commercially available, chemically-defined, and xeno-free adhesion coating, in conjugation with a replating step. Secondly, we integrated the xeno-free differentiation method with metabolic purification/enrichment approaches by optimizing the supplementation of Plurisin1 and Glu^−^Lac^+^ medium. Our results showed that the xeno-free differentiation platform integrated with the two purification/enrichment steps was able to generate structurally integrated CM sheets with a yield of 2.6 hiPSC-CMs per each hiPSC.

Our study has limitations since to develop our xeno-free platform we focused on differentiation efficiency and CM output, while maintaining CM sheet integrity. One of the key limitations of our study is the lack of a teratoma assay, which is required to assess the efficiency of both metabolic purification/enrichment approaches. Absence of a full characterization of the cardiac sheet content regarding noncardiomyocyte cells should also be noticed, in particular, presence of vascular and stromal cells, which would provide additional information about functionality. In addition, functional studies performed were limited and a more detailed maturity profile of the hiPSC-derived CMs would add further insights, in particular, measurement of the expression of key important calcium-handling proteins, assessment of metabolic shifts, and mitochondria maturation.

One of the critical steps of our xeno-free platform is the replating of the cardiac sheet before severe detachment occurs, since detachment results in lower CM output and loss of CM functionality. Partially detached cardiac tissues usually ended up folding upon themselves, creating significant cell masses that stop contracting and lose viability after a few days in culture. The process of replating fully reestablished the cardiac sheet at day 6, after cardiac commitment reached the CPCs stage [32], essential to increase CM output and differentiation efficiency. Burridge et al. also observed detachment of differentiating cells when xeno-free coatings were used, except in the presence of human recombinant laminins, which are still a costly coating solution [18]. Both hESC and derived CMs express similar integrin subtypes [17, 19]. Cell binding to Matrigel and laminin is mostly mediated by *α*6*β*1 integrins, while binding to vitronectin and Synthemax is mediated by *α*V*β*5 integrins [17], which have a 10-fold lower binding affinity [16]. Loss of attachment was mostly seen during or after CHIR and IWP4 exposure, which can be connected with changes in integrin level expression and cellular stress that might occur during commitment towards cardiac mesoderm [18].

In addition to the replating step, we integrated the xeno-free differentiation platform with two steps of purification/enrichment using metabolic strategies. Both methods used supplementation with PluriSin1 and Glu^−^Lac^+^, previously showed to prevent independently, the formation of teratomas [27, 28]. Ben-David et al. reported that mixed cultures of undifferentiated and differentiated hPSCs supplemented with 20 *μ*M of PluriSin1 for two consecutive days prevented teratoma formation [27, 33]. Similarly to what we observed for hiPSCs, Tohyama et al. reported that 24 to 48 h of Glu^−^Lac^+^ medium was enough to fully compromise the viability of mouse PSCs and noncardiomyocyte human cell lines, such as HepG2 and primary cultured peripheral lymphatic cells [28]. For human PSC-derived embryoid bodies, authors used Glu-Lac+ for 6 days to maximize the percentage of CMs obtained [28]. Embryoid body differentiation is known to originate a mixed population of differentiated cells, resulting in poor yields and multiple cell type contaminants [34]. For our differentiation platform, we used an efficient monolayer differentiation protocol relying in the dominant role of WNT signaling [35]. Consequently, higher efficient differentiation should result, hypothetically, in a lower percentage of undifferentiated hiPSCs, which are also more exposed to the metabolic modulators when cultured in a monolayer environment [36–38]. In addition, we gave more emphasis to cell sheet integrity, instead of tuning the Glu^−^Lac^+^ method towards particularly high CM purities. In fact, long culture times with Glu^−^Lac^+^ can lead to excessive loss of viability of noncardiomyocyte cardiac cells that are of extreme importance to provide support for the maintenance and further maturation of the derived hiPSC-CMs [20, 28, 39]. To that effect, our optimal condition was defined as 20 *μ*M PluriSin1 and Glu^−^Lac^+^ up to 48 h. When using replating, differentiation cultures were more sensitive and toxicity/detachment was observed if the application of these methods surpassed the duration of 24 h. SCD1 activity, inhibited by PluriSin1, is also important to other cell types and has a role in the inhibition of neonatal cardiomyocyte apoptosis and reactive oxygen species generation [40]. Therefore, observed sensitivity of replated cells to PluriSin1 can be related to inability to counteract the stress caused by the manipulation of the cells, which in conjugation with lower cellular densities—higher exposure—led to cellular death and consequent cell sheet disruption when exposed to PluriSin1 for consecutive days. Nevertheless, coupling both methods has the potential to generate clinically safe hiPSC-CM sheets. Confirmation of teratoma formation prevention and necessary optimizations, relying on the tunability of the purification methods selected, should be performed for each specific application.

In addition to the purification methods explored in this work, other metabolic strategies for purification and enrichment of hiPSC-derived cardiac cultures, such as the use of high concentrations of L-Alanine, might be explored [41]. Moreover, coupling metabolic purification and enrichment methods with metabolic maturation, such as shifting CM metabolism towards fatty acid oxidation, can provide multiple synergistic advantages [42–44].

As mentioned before, the maintenance of cardiac sheets integrity is important for their long-term maturation. A maturation step is essential for the development of reliable disease models and platforms for drug screening, since immature hiPSC-CMs, lacking the biochemical structure and electrophysiology of adult CMs, can lead to wary outcomes [43, 45–47]. We previously showed that long-term cultures, up to 120 days, significantly improve structural maturation of CM sheets [20]. Other maturation approaches, such as electrical stimulation of hiPSC-CMs, might promote faster maturation, originating CMs with improved electrophysiological and calcium transient profiles [48–52].

## 5. Conclusions

In conclusion, we successfully implemented a xeno-free cardiac differentiation platform by introducing and optimizing a replating step, which resulted in structurally integrated cardiac sheets, increased differentiation yields and increased CM outputs. In addition, we integrated the xeno-free differentiation platform with two versatile purification and enrichment steps, previously shown to prevent teratoma formation, essential when hiPSC-CMs are intended for applications in regenerative medicine. Our platform is compatible with long-term culture, or other maturation methods, which can facilitate further improvements in the development of reliable disease models and drug screening platforms.

## Data Availability

The data supporting the findings of this study are available upon request.

## Supplementary Materials

Supplementary Materials Supplementary Table S1: primer pairs used for real-time PCR. Supplementary Figure S1: optimization of cardiomyocyte differentiation and replating using different culture systems. Supplementary Figure S2: purification and enrichment steps optimization. Supplementary Video S1: time-lapse of the first 7 days of cardiac differentiation using the Wnt signaling protocol. Supplementary Video S2: cardiac sheet obtained at day 15 using 6 *μ*m of CHIR to induce cardiac differentiation of hiPSC growing in Matrigel-coated plates. Supplementary Video S3: representative example of cellular and cardiomyocyte sheet detachment from plates with xeno-free coatings. Supplementary Video S4: detachment observed at day 15 by the naked eye for hiPSC in Synthemax-coated plates differentiated using 6 *μ*M of CHIR and with or without exposure to the purification and enrichment steps. Supplementary Video S5: replating 1 : 1 at day 6 allowed the reconstitution of the CM sheets with contraction visible by the naked eye at day 15.
