# Supplementary material for: Xeno-Free Integrated Platform for Robust Production of Cardiomyocyte Sheets from hiPSCs
Source: Stem Cells Int. 2022 Nov 23;2022:4542719. doi: 10.1155/2022/4542719 (PMC9712013; doi:10.1155/2022/4542719)
Supplement: Supplementary Materials — Supplementary Table S1: primer pairs used for real-time PCR. Supplementary Figure S1: optimization of cardiomyocyte differentiation and replating using different culture systems. Supplementary Figure S2: purification and enrichment steps optimization. Supplementary Video S1: time-lapse of the first 7 days of cardiac differentiation using the Wnt signaling protocol. Supplementary Video S2: cardiac sheet obtained at day 15 using 6 μm of CHIR to induce cardiac differentiation of hiPSC growing in Matrigel-coated plates. Supplementary Video S3: representative example of cellular and cardiomyocyte sheet detachment from plates with xeno-free coatings. Supplementary Video S4: detachment observed at day 15 by the naked eye for hiPSC in Synthemax-coated plates differentiated using 6 μM of CHIR and with or without exposure to the purification and enrichment steps. Supplementary Video S5: replating 1 : 1 at day 6 allowed the reconstitution of the CM sheets with contraction visible by the naked eye at day 15. [file 4542719.f1.zip › Supp Info.pdf]

Supplementary Information

Xeno-free integrated platform for robust production of cardiomyocyte sheets from hiPSCs

Dias *et al*.

Table S1. Primer pairs used for real-time PCR.

Melting temperatures (Tm), amplicon size and primer specificity were estimated using Primer Blast.

Gene	Primers (5’ > 3’)	bp	Tm (°C)	Amplicon Size
T	Fwd: CTATTCTGACAACTCACCTGCAT	23	60.0	146
	Rev: ACAGGCTGGGGTACTGACT	19	61.9	
MIXL1	Fwd: TACCCCGACATCCACTTGCG	20	62.2	110
	Rev: CCACTCTGACGCCGAGACTT	20	61.9	
MESP1	Fwd: CTGAAGGGCAGGCGATGGA	19	62.0	83
	Rev: GGGCATCCAGGTCTCCAACA	20	61.9	
NKX2.5	Fwd: CCAAGGACCCTAGAGCCGAA	20	61.0	77
	Rev: GTCCGCCTCTGTCTTCTCCA	20	61.3	
ISL1	Fwd: GCGGAGTGTAATCAGTATTTGGA	23	60.1	102
	Rev: GCATTTGATCCCGTACAACCT	21	60.4	
GAPDH	Fwd: ACAACTTTGGTATCGTGGAAGG	22	60.2	101
	Rev: GCCATCACGCCACAGTTTC	19	61.7	

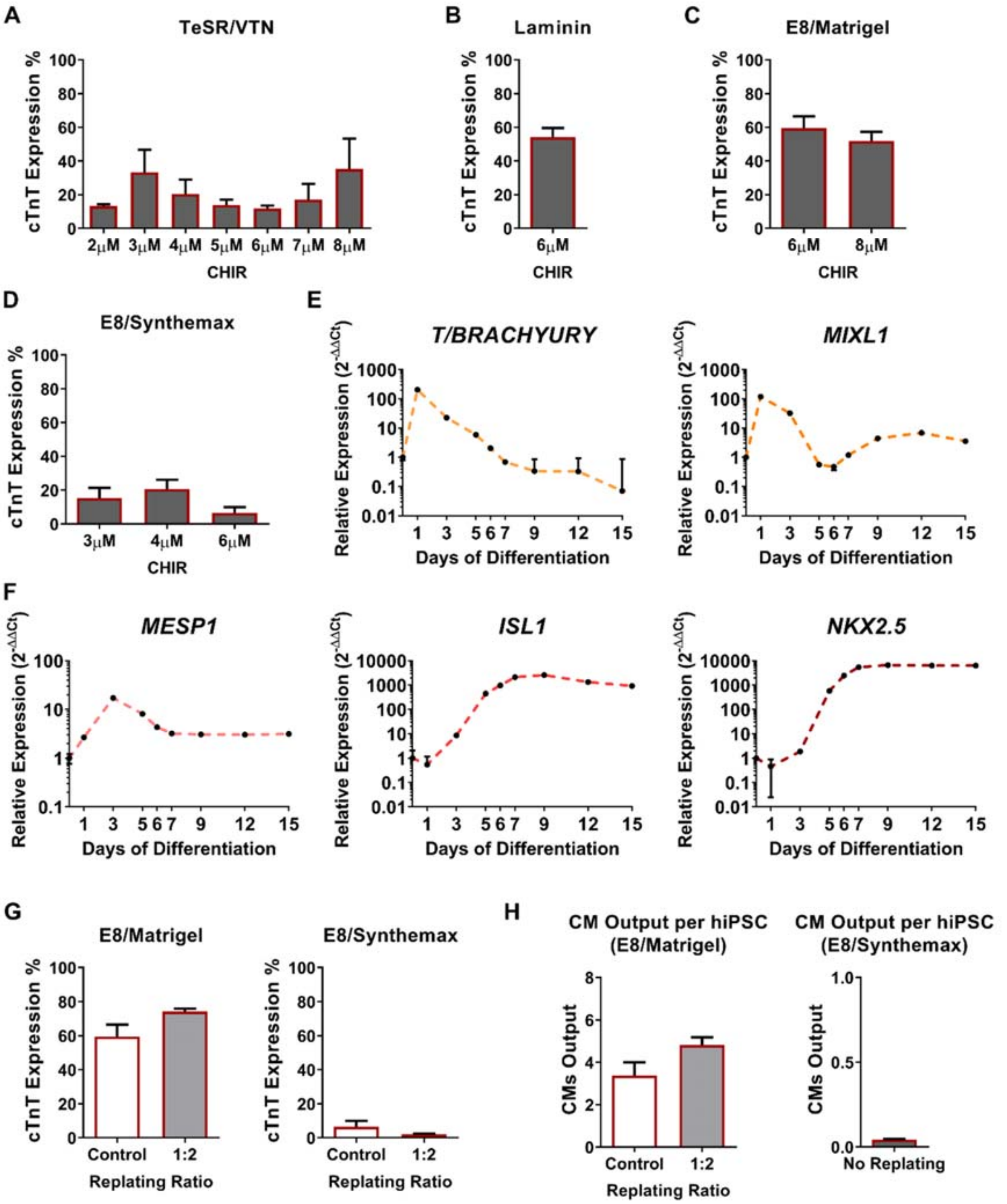

Figure S1. Optimization of cardiomyocyte differentiation and replating using different culture systems. (A) Screening of initial CHIR concentration impact on differentiation efficiency at day 15 by flow cytometry using hiPSCs cultured in TeSR and Vitronectin. Sensibility to higher CHIR concentrations resulting in significant cell detachment was observed. Error bars, SEM, n=3. **(B)** TeSR/Laminin had comparable efficiency to TeSR/Synthemax and TeSR/Matrigel (Fig. 1). Error bars, SEM, n=5. **(C)** E8 medium resulted in cTnT % positive cells similar to TeSR when Matrigel was used (Fig. 1).

Error bars, SEM, n=6 for 6 µM, n=2 for 8 µM. **(D)** While hiPSCs in E8/Synthemax usually detached or did not efficiently differentiate. Error bars, SEM, n=2 for 3 µM, n=3 for 4 µM, n=6 for 6 µM. **(E, F)** Relative expression profiles for mesendoderm and cardiac mesoderm markers during the 15 days of hiPSC cardiac differentiation. Error bars, SD, n=3. **(G)** Replating 1:2 maintained cTnT % for E8/Matrigel with non-significant statistical differences (Welch's t-test) compared with the control (same as Fig. S1C 6 µM). Error bars, SEM, n=6. For E8/Synthemax replating was unable to revert low efficiency and detachment (control same as Fig. S1D 6 µM). Error bars, SEM, n=4 for Replating. **(H)** Cardiomyocyte (CM) output for each system is concordant with the differentiation efficiency observed (Fig. S1G) with E8/Matrigel, control and Replating 1:2, having a comparable output to TeSR/Matrigel (Fig. 2E). Error bars, SEM, n=3 for E8/Matrigel Control, n=6 for E8 Replating 1:2, n=3 for E8/Synthemax.

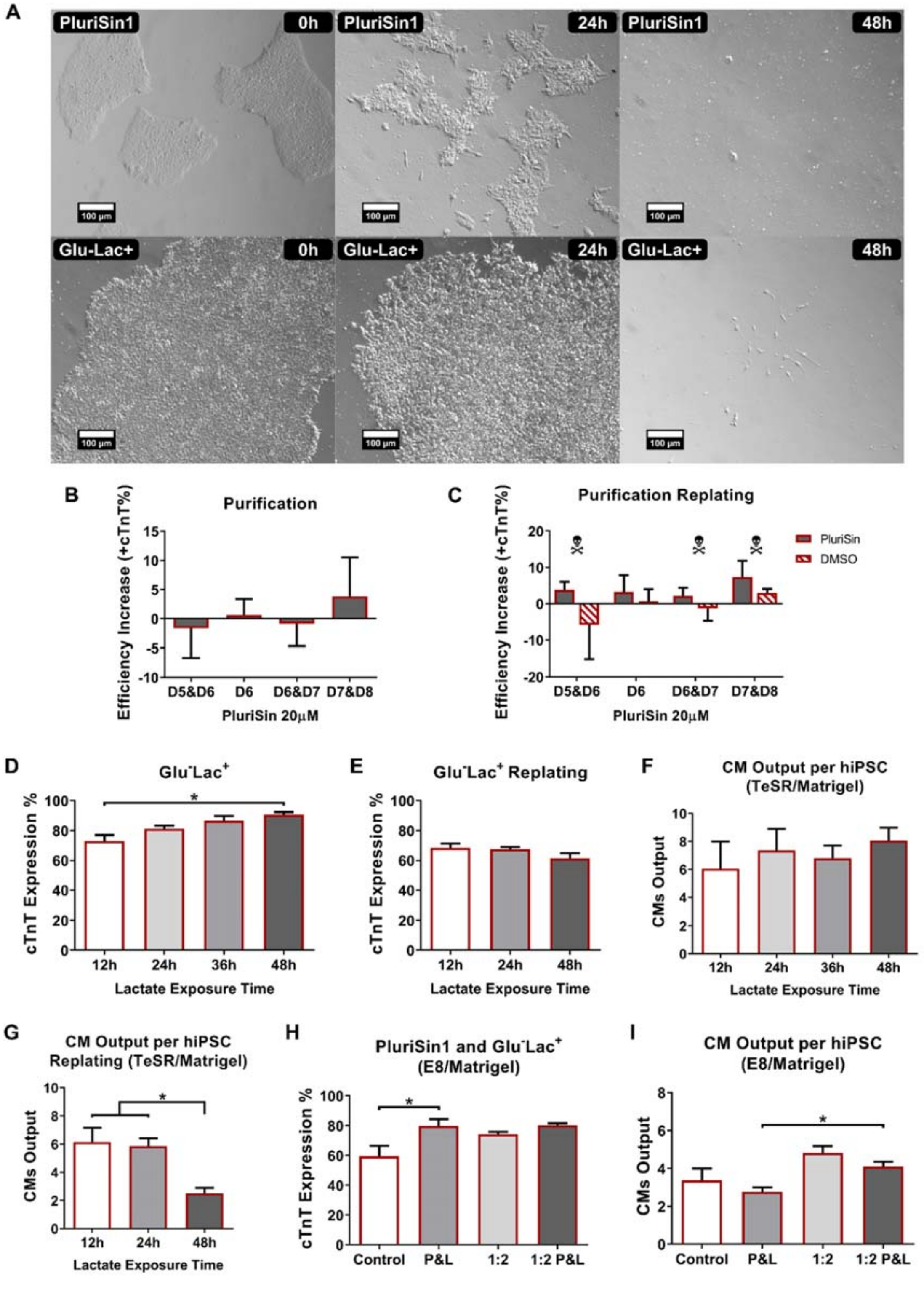

Figure S2. Purification and enrichment steps optimization.

(A) Effect of PluriSin1 (two times 20 µM) and Glucose-free media supplemented with Lactate (Glu^{-}Lac^{+}) for 48 h in hiPSC cultures. Both approaches eliminated all hiPSCs in 48

h. Image of PluriSin1 48h shows only some cell debris, while Glu^{-}Lac^{+} 48 h shows only cells with a fibroblast-like phenotype. Cells were maintained 4 more days in mTeSR1 after the 48 h exposure to guarantee that hypothetic hiPSCs were not present. Scale bar 100 µm. **(B, C)** Addition of PlusriSin1 had no significant effect on cardiomyocyte differentiation efficiency without replating (B), while often showing to lead to cell death (☠) upon replating when an exposure of two consecutive days was used at day 5 and 6, day 6 and 7 or day 7 and 8 (after replating) (C). Error bars, SEM, n=6 for D6 without replating, n=10 for D6 with replating, n=4 for all other days with and without replating. DMSO was used as vehicle control, n=2. **(D)** Exposure to Glu^{-}Lac^{+} increased the percentage of cTnT positive cells at the end of differentiation in an exposure time dependent manner. Error bars, SEM, n=2. **(E)** For replating (1:3), exposure to Glu^{-}Lac^{+} for 48 h revealed to decrease CM sheet stability leading to cardiomyocyte detachment. Error bars, SEM, n=5 for 12 h and 24 h, n=4 for 48 h. **(F)** Cardiomyocyte (CM) output was not significantly different for different times of exposure to Glu^{-}Lac^{+}. Error bars, SEM, n=2. **(G)** On the other hand, for replating, 48 h had a significant impact on output due to increased cell detachment. Error bars, SEM, n=5 for 12 h and 48 h, and n=2 for 48 h. **(H)** For E8/Matrigel, simultaneous use of both PluriSin1 and Glu^{-}Lac^{+} (P&L) significantly enriched the culture with cardiomyocytes increasing cTnT expression compared with control. Exposure to Glu^{-}Lac^{+} for conditions without replating (control) was 48 h and for replating conditions 24 h. Error bars, SEM, n=3 for P&L and Replating 1:2 P&L, n=6 for Control and Replating 1:2. **(I)** CM output for E8/Matrigel significantly increased with replating 1:2 when using P&L. Error bars, SEM, n=6 for Replating 1:2 and n=3 for other conditions. *p-value<0.05 (Welch's t-test).

Video S1. Time-lapse of the first 7 days of cardiac differentiation using the Wnt signaling protocol. Each frame was obtained every 2 min for approximately 24 h. Video compiled at 40 frames per second.

Video S2. Cardiac sheet obtained at day 15 using 6 µM of CHIR to induce cardiac differentiation of hiPSC growing in Matrigel-coated plates. Scale bar: 100 µm.

Video S3. Representative example of cellular and CM sheet detachment from plates with xeno-free coatings. In the video, the CM sheet folded upon itself. Scale bar 100 µm.

Video S4. At the bottom (middle row of 12 well-plate), two replicates of hiPSC in Synthemax-coated plates differentiated using 6 µM of CHIR, with both showing severe detachment at day 15. At top, two replicates of hiPSC in Synthemax-coated plates

differentiated using 6 µM of CHIR and exposed to the purification and enrichment steps, further increasing cellular detachment at day 15.

Video S5. Replating 1:1 at day 6 allowed the reconstitution of the CM sheets with contraction visible by the naked eye at day 15. At the bottom (middle row of 12 well-plate), two replicates replated from hiPSC in Synthemax-coated plates differentiated using 6 µM of CHIR. At top, two replicates replated from hiPSC in Synthemax-coated plates differentiated using 6 µM of CHIR and exposed to the purification and enrichment steps.
